# How to Achieve a Healthy City: a Scoping Review with Ten City Examples

**DOI:** 10.1007/s11524-023-00798-9

**Published:** 2023-12-18

**Authors:** Abbas Ziafati Bafarasat, Ayyoob Sharifi

**Affiliations:** 1 School of the Built Environment, Oxford Brookes University, Oxford, UK; 2 The IDEC Institute & Network for Education and Research On Peace and Sustainability (NERPS), Hiroshima University, Higashi-Hiroshima, Japan; 3 School of Architecture and Design, Lebanese American University, Beirut, Lebanon

**Keywords:** Healthy cities, Public health, Urban planning, Sustainable development, Urban health, World Health Organization (WHO)

## Abstract

This scoping review of the literature explores the following question: what systematic measures are needed to achieve a healthy city? The World Health Organization (WHO) suggests 11 characteristics of a healthy city. Measures contributing to these characteristics are extracted and classified into 29 themes. Implementation of some of these measures is illustrated by examples from Freiburg, Greater Vancouver, Singapore, Seattle, New York City, London, Nantes, Exeter, Copenhagen, and Washington, DC. The identified measures and examples indicate that a healthy city is a system of healthy sectors. A discussion section suggests healthy directions for nine sectors in a healthy city. These sectors include transportation, housing, schools, city planning, local government, environmental management, retail, heritage, and healthcare. Future work is advised to put more focus on characteristic 5 (i.e., the meeting of basic needs for all the city's people) and characteristic 10 (i.e., public health and sick care services accessible to all) of a healthy city.

## Introduction

### Research Question

**Fig. 1:**
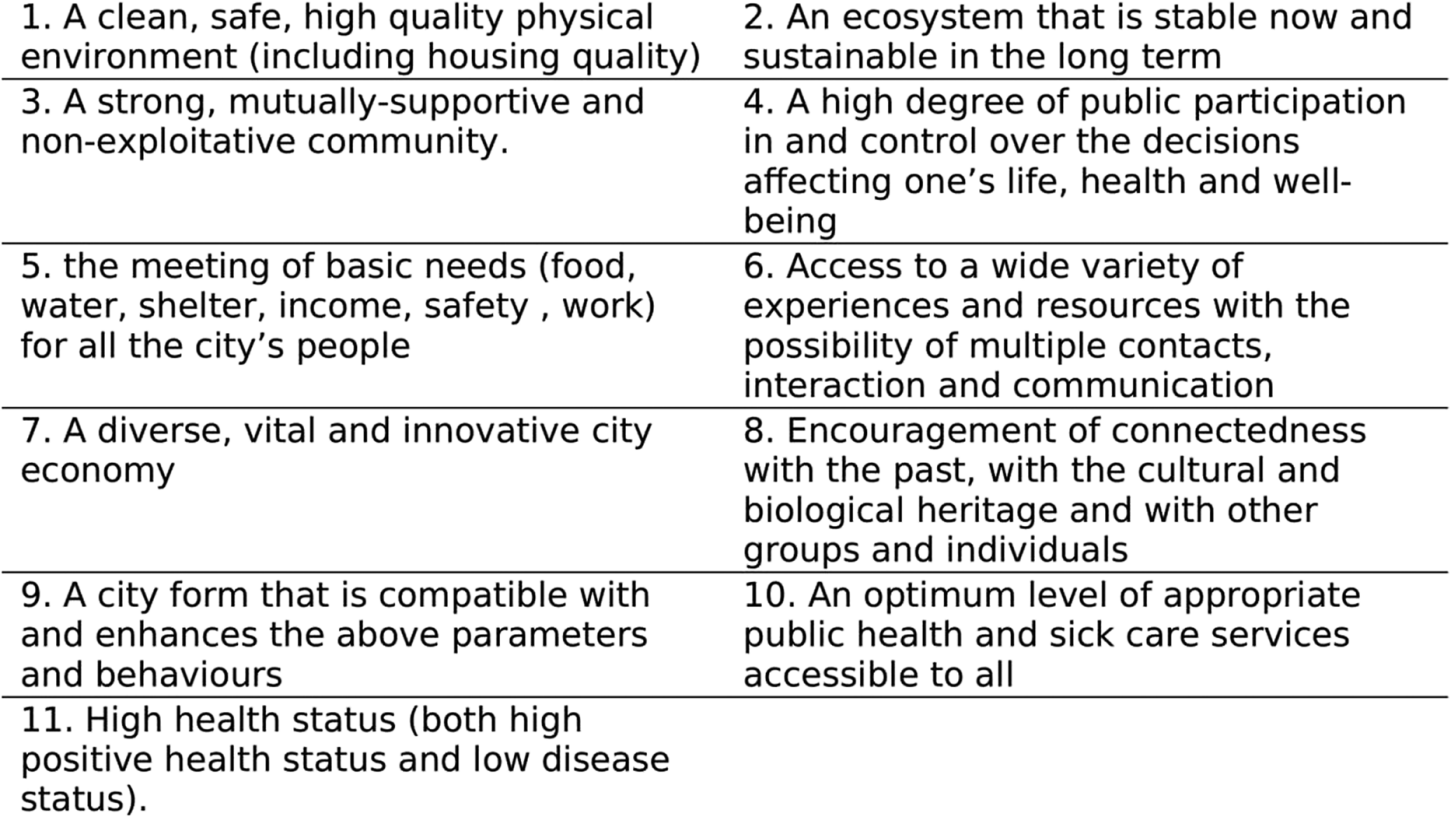
The 11 characteristics of a healthy city according to the WHO [4]

A healthy city involves more than healthy homes, streets, and parks. Healthy directions in different sectors, from schools to healthcare, make up a healthy city as a whole system. Knowledge about this system and its interconnections is scattered. It is less accessible to the diverse actors shaping cities [1–3]. This study is a scoping literature review to explore: what systematic measures are needed to achieve a healthy city? The World Health Organization (WHO) suggests that a healthy city involves 11 characteristics (Fig. 1). This study therefore seeks to explore what systematic measures are needed to achieve the 11 characteristics of a healthy city.

### Healthy City

**Fig. 2:**
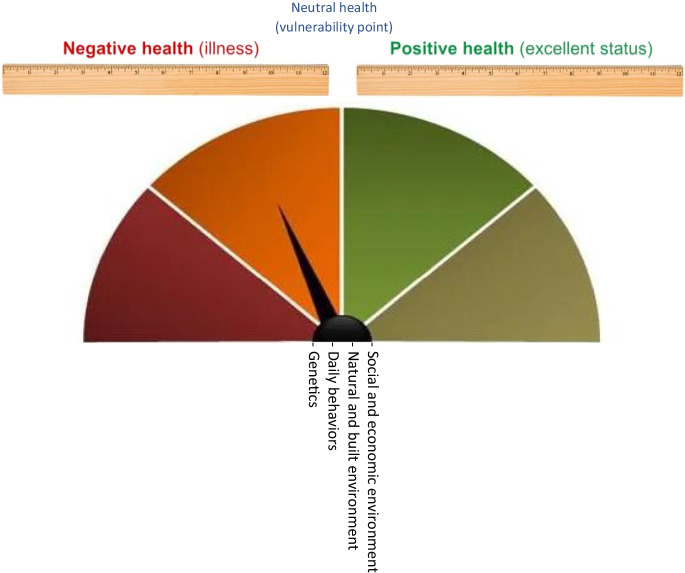
The health spectrum (Authors)

Health is mostly described as the absence of disease [5]. However, according to the constitution of the WHO, “Health is a state of complete physical, mental and social well-being and not merely the absence of disease or infirmity” (WHO, [6], 1). Living conditions and genetics are health determinants. Living conditions consist of (I) social and economic environment, (II) physical (built and natural) environment, and (III) daily behaviors [7]. Living conditions and genetics determine health status on a spectrum that is displayed in Fig. 2. Points on the spectrum that are further on the positive health side indicate more health assets, such as clean air, income, and active lifestyles. Therefore, they indicate more resistance or distance to illness (negative health). WHO’s constitutional definition of health refers to positive health.

It is often said that living conditions are more important than genetics in determining one’s health [8]. Currently, the living conditions of most people worldwide are urban [9]. Urban living conditions tend to move us away from positive health toward illness (Fig. 1) by inadequate public services, environmental pollution, poor housing, stress, sedentary lifestyle, and alike [10]. Efforts have been made since the mid-nineteenth century to tackle this trend. Modern-day public health traces its roots to the *Health in Towns Commission* established by the British government in 1843. The WHO program of Healthy Cities is the global, systematic culmination of these efforts [11]. WHO initiated the Healthy Cities Program in 1986 to promote health as the guiding principle of sectoral policies and urban plans in participating cities [4]. In the first series of WHO Healthy Cities Papers, a healthy city was defined as:

*“one that continually creates and improves its physical and social environments and expands the community resources that enable people to mutually support each other in performing all the functions of life and developing to their maximum potential.”* [12], 41).

This ground-setting WHO publication [12] identified 11 characteristics of a healthy city. These characteristics (Fig. 1) interconnect citizens’ physical, economic, and social health and integrate them with the health of animals, plants, and the environment. Subsequent studies in this field are more focused but less holistic [13]. For example, the planetary health perspective focuses on situating human health in the well-being of the earth including living and nonliving systems [14]. However, it is less comprehensive of discussions about economic health. Some 1400 municipalities worldwide that are members of the WHO Healthy Cities Network aim to achieve the 11 characteristics [15, 16]. Many other cities are inspired by these characteristics but wonder how to achieve them with a systematic approach [1].

To explore systematic measures to achieve the 11 characteristics of a healthy city, we undertake a rigorous scoping review in line with the methodological guidelines of JBI (Joanna Briggs Institute) [17] and PRISMA-ScR [18]. This is explained in the Section “Research Method.” The Section “Results and Discussion” presents and discusses the findings of this study. It presents, in 29 themes, systematic measures to achieve the characteristics of a healthy city. The implementation of some of these measures is illustrated by city examples from the literature. The Section “Results and Discussion” discusses the findings by suggesting healthy directions for nine sectors in a healthy city and identifying gaps in the knowledge and practice of healthy cities. The Section “Conclusions” provides the conclusions of this study.

## Research Method

### Systematic Scoping Review

This is a scoping review of the literature. Scoping reviews are a form of knowledge synthesis that usually have three main features as follows:

- Scoping reviews begin with broad and topic-focused questions.
- Scoping reviews apply a range of study designs to comprehensively summarize and synthesize evidence, for instance in a tabular list.
- Scoping reviews seek to inform policy and practice. They might also provide direction to future research [17, 19, 20].

**Fig. 3:**
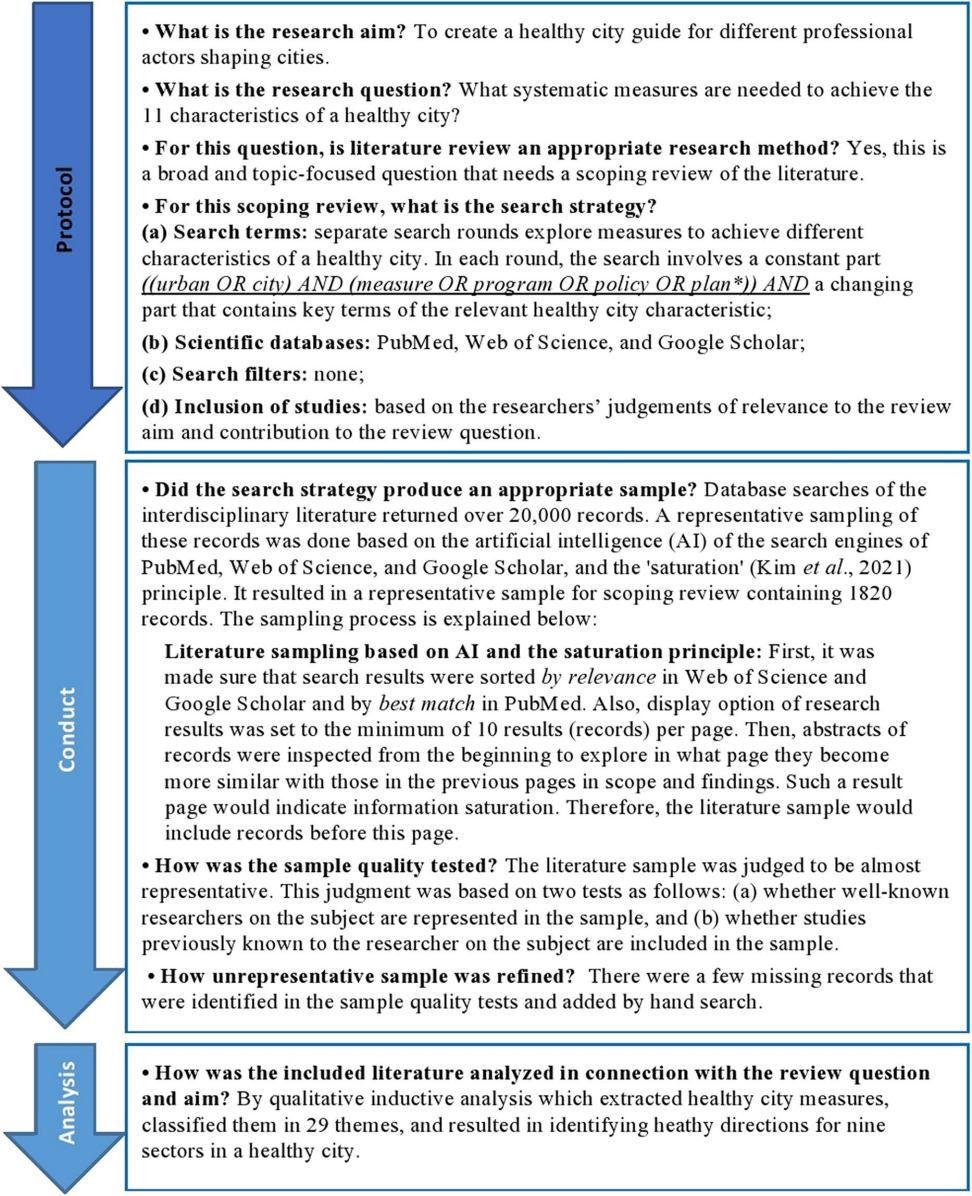
Protocol, conduct, and analysis of this scoping review (Authors, based on: [17, 18]

**Fig. 4:**
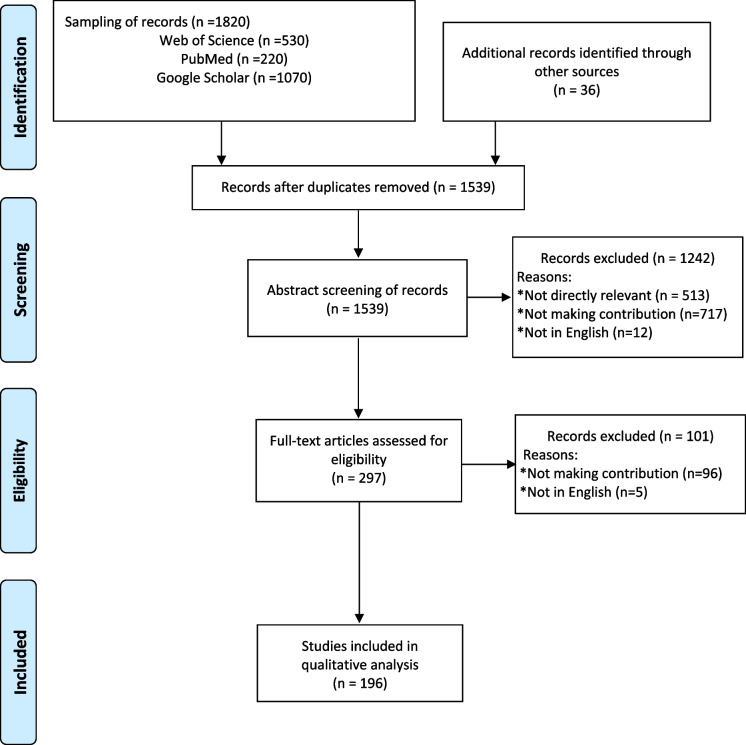
PRISMA-ScR flow diagram of this scoping review (Authors, based on: [18, 21]

This scoping review follows a systematic design. This means that its aim and question, inclusion criteria, and analysis method were specified in advance and documented in a protocol. The protocol, conduct, and analysis in this scoping review (Fig. 3) are in line with two globally recognized methodological sources for evidence synthesis. The first source is the JBI methodological manual [17] which includes chapters on systematic reviews and scoping reviews [21], among others. The second methodological source, which is a scoping review supplemental to the JBI methodological manual, is the PRISMA-ScR guidelines. PRISMA-ScR guides reporting of literature identification, screening, eligibility, and inclusion in systematic scoping reviews [18]. Figure 4 provides a PRISMA-ScR report for this scoping review.

### Inductive Content Analysis

An information extraction template was developed in Microsoft Word to collect evidence from records included in the analysis. Selected contents from records were entered into the template to undergo qualitative analysis and produce shorter contents as healthy city measures. When checking healthy city measures collected from the first literature record, the measures were divided into several themes depending on their focus. While checking healthy city measures collected from the next literature record, it was examined if the measures could be classified under existing themes or should be classified as distinct ones. In other words, new themes were identified if the measures could not be subsumed to one previously identified theme [22]. At the end of this process, there were 29 themes. They resulted in identifying healthy directions for nine sectors in a healthy city.

In other words, the analysis stage of this review study was conducted manually using an inductive qualitative content-analysis method [22]. This method allows extracting insights from the literature more comprehensively and without preconceived standards and categorization [23]. In the case of our study, this means without previous assumptions about the themes. Despite these benefits, the method involves a time-consuming process and certain levels of subjectivity [24].

#### Choices of City Examples

This scoping review provides ten city examples illustrating the implementation of some healthy city measures. These examples include Freiburg, Greater Vancouver, Singapore, Seattle, New York City, London, Nantes, Exeter, Copenhagen, and Washington, DC. Seven of these city examples appeared in the included literature, but Exeter, Washington, DC, and Copenhagen were identified in Google and Google Scholar searches by using the key terms of measures of healthy city characteristics 8, 9, and 10 to explore city examples. The examples are provided as a guide to the application of some healthy city measures rather than best practices. In fact, not many cities meet the definition of a healthy city given at the beginning of this study [25]. However, most of the ten examples appear in the literature as progressive cities in health promotion.

## Results and Discussion

### Overview of the Literature

The 196 records included in the full-text analysis belonged to a wide range of disciplines, such as public health, city planning, built environment, transportation studies, political science, public administration, agriculture and life sciences, environmental management, human geography, sociology, and economics. However, city planning was more represented in the literature sample.

Regarding geographical representation, North America and Europe were most represented, and Africa was least represented. The scale of studies ranged from urban neighborhoods to cities and the regional scale. In terms of target bodies to act on the findings of the studies, there was a focus on local governments and departmental sectors beyond the health sector.

### Healthy City Measures and their Examples

Tables 1, 2, 3, 4, 5, 6, 7, 8, 9 and 10 collate and summarize the review results in a systematic list of measures to achieve the characteristics of a healthy city. The characteristic 11 (high health status) is not covered standalone because it is the overall outcome of the other 10 characteristics. The healthy city measures provide a systematic toolkit for diverse professional actors shaping our cities. The measures appear under 29 themes, such as healthy transportation (Table 1). The tables are followed by 10 real city examples for the application of some of the healthy city measures.

**Table 1:** Measures to achieve “A clean, safe, high quality physical environment (including housing quality)” (characteristic 1 of a healthy city)

**Healthy transportation:** a coherent system of walking, cycling, and public transport in which: 1. There are continuous cycling paths or lanes 2. Network layout supports walking for people of different abilities (e.g., smaller urban blocks , less frequent dead-ends) 3. Sidewalks follow climate-sensitive and inclusive design 4. Public transport is accessible in physical, economic, and cultural aspects **Integrated impact assessment:** appraising and mitigating different impacts (e.g., carbon emission, natural hazards, crime) of development in integration between policy and project levels. This means that where the policy level is in principle against some type of development, impact assessment of such development projects should consider a “no or without project option” **Parks:** designing public parks that encourage visitation, physical activity, and social interaction **Smoke-free environments:** smoke-free designations beyond indoor spaces to also include selective footpaths, small parks, public transport stops, and open spaces of healthcare facilities **Adaptable housing:** 1. Designing housing that is adaptable to changing (e.g., family size, aging) needs. Housing adaptability without construction changes depends on the size, location, and interrelation of spaces in the house. Adaptability by changes to floor layout also depends on permitted developments rights 2. Providing education and resources for households to improve their home environments and tackle indoor health risks like lead, injury, and poor ventilation **Complete neighborhoods: **providing at the neighborhood level the full range of day-to-day services and facilities	[26–44]

**Table 2:** Measures to achieve “An ecosystem which is stable now and sustainable in the long term” (characteristic 2 of a healthy city)

**Regional collaboration:** joint action between neighboring localities to: 1. Contain urbanization and protect natural resources 2. Promote low carbon mass transit 3. Integrate waste management **Integrated impact assessment:** reflecting impact assessment of policies and plans in impact assessment of projects (e.g., alternative scenarios for development) **Eco-design:** 1. Biodiversity-sensitive design. One example is islands or strips of habitat in cities containing native plants, hollow logs, ephemeral streams, etc 2. Energy-sensitive design. This means that it minimizes the need for travel, and applies nature-based solutions to control indoor temperature 3. Water-sensitive design. This means that it minimizes the need for water and applies nature-based solutions to recycle water 4. Design that mixes activities that have different patterns of resource use to support residual flow consumption	[49–51, 52, 53, 54, 163, 164, 165, 166, 167, 168]

**Table 3:** Measures to achieve “A strong, mutually- supportive and non-exploitative community” (characteristic 3 of a healthy city)

**Community-sensitive housing:** 1. Housing regeneration that involves minimum community displacement but increases public space and services throughout the site 2. Relocating or consolidating land-intensive facilities to free up space for the development of affordable housing 3. Planning a sensible mix of housing tenures (private tenancy, public tenancy, owner occupancy) at the neighborhood level 4. Planning a sensible mix of housing types (e.g., single-family houses, duplexes, apartment buildings) at the neighborhood level 5. Avoiding “poor doors” and “segregating design” (e.g., separate play areas) in affordable housing projects 6. Planning of ethnic and cultural mix in large-scale public housing **Urban commons:** 1. Public spaces that help women and minorities thrive (e.g., express their identity, do business) and connect 2. Public spaces that serve different ages, abilities, and interests 3. Community halls that through cultural, social, and physical activities develop relationships between young people and seniors to pass on values 4. Community ownership of local assets such as wind farms, bookstores, retail stores, and football clubs **Community-sensitive schools:** schools’ policy should promote feeder schools that typically keep classmates from the whole community together from the first grade through high school. It should also promote parental and student participation in school management	[25, 40, 59–71]

**Table 4:** Measures to achieve “A high degree of participation in and control over the decisions affecting one's life, health and well-being” (characteristic 4 of a healthy city)

**Self-governing neighborhoods:** 1. Help communities establish associations/forums 2. Provide communities with the power to decide about the type of housing, amenities, and services that they need **Autonomous local government:** local government should have freedom from central interference but the ability to effect particular outcomes in consultation with citizens **Participative local government:** directly engaging people in municipal decisions through consultation methods that: 1. Encourage community organizing by seeking policy advice from community health groups, neighborhood forums, etc 2. Inform citizens (e.g., citizens juries, public hearings) 3. Include large numbers of people (e.g., surveys, referenda) 4. Seek citizens’ views before, during, and after interventions (e.g., citizens panels, surveys) 5. Reach out to particular and underrepresented groups (e.g., focus groups, the trusted advocates model) 6. Involve on-site graphical consultation (e.g., charrettes) 7. Help consultation seekers look through the lens of local community(e.g., validity test in landscape analysis).	[40, 68, 75–83]

**Table 5:** Measures to achieve “The meeting of basic needs (food, water, shelter, income, safety, work) for all the city's people” (characteristic 5 of a healthy city)

**Housing policy that is empowering:** 1. Affordable housing projects should consider all basic needs (e.g., clean water, healthy food, employment, public transport) of residents rather than only the need for housing 2. Assistance for the homeless should follow the Housing First model rather than the Treatment First model In the Treatment First model, homeless people should first qualify for rent assistance by addressing their lifestyle problems while in collective, temporary, and supervised accommodations. By contrast, the Housing First model sets no prerequisites for rent assistance as it considers stable housing as the foundation for life improvement **Localized food and people-centered retail:** 1. Preserving farmlands on the urban fringe and supporting farmers’ markets 2. Defining multifunctional agriculture in cities as a legitimate and desirable category of land use 3. Encouraging the emergence of small service establishments and fresh food stores in residential areas 4. Enabling street vending as a legitimate livelihood—thus capitalizing on its ability to generate employment and revenue and to provide goods at low prices in convenient locations	[38, 62, 75, 87–97]

**Table 6:** Measures to achieve “Access to a wide variety of experiences and resources with the possibility of multiple contacts, interaction and communication” (characteristic 6 of a healthy city)

**Zoning that characterizes**: 1. Zoning needs to ensure that different areas in and around the city maintain their particular character and specific relationship with the environment. This zoning policy is reflected in the “rural-to-urban transect” model which proposes a series of zones from sparse rural farmhouses (T1) to the dense urban core (T6) 2. Zoning needs to promote a polycentric urban structure, for instance, by allocating main employment floor space to several transit hubs **Complete neighborhoods:** planning complete neighborhoods that provide walking access to most urban functions, such as shops, schools, workplaces, parks, cultural facilities, and public transport stations **Public space that is enabling:** 1. Public space should support all the senses, not only the visual 2. Public space should cater for day and night experiences 3. Public space should allow different people to act out their own style of social life but ultimately bring them into contact with crowds of strangers 4. Open public space (e.g. parks, woodlands, public beaches) should not limit access by fences and gates **Healthy transportation:** 1. Seamless integration between all modes of moving in and around the city (e.g., park and ride facilities, single transport cards, bike rental near stations) 2. Supporting wayfinding, particularly for visitors the visually impaired and people with dementia, by measures such as frequent street signposting 3. Public transport concession for the elderly 4. Disability-friendly streets	[29, 101–111]

**Table 7:** Measures to achieve “A diverse, vital and innovative city economy” (characteristic 7 of a healthy city)

**Regional collaboration:** integrating economic policies and land use plans of adjoining cities to foster a cluster of economic activities **Localized business:** 1. Regulating the size and minimizing the quantity of formula businesses 2. Enabling case-by-case exceptions to zoning ordinances to support commercial lots that use and strengthen community resources, businesses that are employment and purchasing partners to the city, and home-based entrepreneurship 3. Provision of land for consistent growth of job and housing **Innovative city center:** city center policy might integrate a whole package of science, business, life, and entertainment to foster innovation in economy. For example, a city center could bring together: 1. Ungated university campuses and living labs (which move research out of laboratories into public space) 2. Commercial and office spaces 3. Startup co-working and co-living spaces 4. A diverse housing stock 5. Street-level retail and nursery 6. Community spaces, etc	[1, 40, 115–120]

**Table 8:** Measures to achieve “Encouragement of connectedness with the past, with the cultural and biological heritage and with other groups and individuals” (characteristic 8 of a healthy city)

**Everyday heritage:** 1. Participative mapping of the city’s cultural (tangible and intangible) and biological heritage 2. Situating heritage in everyday public space. This would enable citizens to see, pass through, smell, hear, or touch their cultural and biological heritage in everyday public life 3. Accommodating education providers, where sensible, in heritage sites and buildings **Integrated preservation:** using mechanisms of protecting heritage that integrate with the requirements of a healthy city as a whole. For example, the transfer of development rights protects heritage sites from development but brings them development income from selling development rights **Cultural city center:** designing the city center like a cultural quarter of memorable places and animating it with street life, festivals, and celebrations **Eco-design:** 1. Design that uses common heritage elements and layouts to integrate different neighborhoods with the urban core 2. Design that protects natural landmarks and enhances eco-built landmarks 3. Design that stimulates playful interaction of people with their environment and with strangers (e.g., green gyms, air mattress-like surface on a sidewalk)	[39, 126–134]

**Table 9:** Measures to achieve “A city form that is compatible with and enhances the above parameters and behaviours” (characteristic 9 of a healthy city)

**Livable density:** urban density is livable that: 1. Acknowledges geographic context (e.g., lower buildings at the shoreline) 2. Integrates with human scale (e.g., ground-level shops and community space in high-rise buildings) 3. Provides contact with green space 4. Provides affordable housing 5. Delivers efficient public services in transportation, healthcare, etc 6. Applies a mix of tools to increase density, i.e., high-rise apartments in strategic locations and gentle increases in neighborhood density 7. Has a polycentric urban structure **Modular form:** a modular (scalable) urban form to absorb future growth or de-growth. Two strategies for modularity are as follows: 1. Neighborhood–based design which enables unit-by-unit changes as the city grows or de-grows 2. Open-ended urban boundaries in natural, physical, and legal terms **Integrated edge:** integrating urban activities that are pushed away to the urban edge (e.g., sewage works, mental institutions, asylum centers) with farming and forestry. This will improve the edge of cities rather than just preserve them from sprawl **Regional collaboration:** coordinating urban form policy between neighboring local authorities	[40, 136–145]

**Table 10:** Measures to achieve “An optimum level of appropriate public health and sick care services accessible to all” (characteristic 10 of a healthy city)

**Community-sensitive healthcare:** 1. Informing the designation of healthcare resources by community health and socio-economic maps 2. Ensuring that there are walk-in clinics in neighborhoods with low-income communities, linguistic minorities, and recent immigrant populations 3. Engaging local communities in setting objectives and designing service and the quality of healthcare **Healthcare-sensitive development:** 1. Providing digital health checks in neighborhood parks 2. Designing a ratio of new housing supply for community-based rehabilitation of persons with disabilities. One example is extra care housing clustered around support services which may be part of wider community amenities 3. Planning urban development with consideration for access to primary and emergency healthcare 4. Designing healthcare services in public housing complexes **Accessible public hospitals:** 1. Building more public hospitals in locations that enable rapid response to medical emergencies in vulnerable districts 2. Providing virtual care in public hospitals to serve patients in remote areas	[40, 148–160]

#### Freiburg, Germany

The university town of Freiburg is known for its promotion of healthy living conditions in multiple dimensions [45]. In this section, we provide an example of its measures to achieve characteristic 1 of a healthy city: “A clean, safe, high quality physical environment”. Freiburg is best known for the development of two model neighborhoods, Rieselfeld and Vauban. Construction of Vauban began in 1998 with community participation, and the first residents arrived in 2001. Vauban offers varied housing options with flexible ground plans for a diversity of families and needs. There are some basic development requirements (e.g., the distance between the houses is defined at 19 m, and the distance between the distinct buildings at 20 m), but the rest is left adjustable [46]. The allotment of small parcels to different developers working with different client groups enabled the creation of flexible housing options based on simple guidelines set by the city authorities. Accordingly, public amenities and institutions were designed to accommodate evolving requirements for both housing and community needs [47].

Vauban offers a comprehensive selection of shopping options for daily needs and an extensive range of professional services. There are some 600 jobs in the neighborhood, all of which can be easily accessed on foot or by bicycle. Vauban benefits from an interconnected network of cycling paths and walking routes that extend throughout the entire area, seamlessly connecting it to the wider city of Freiburg. Arcaded shopping streets support the walking and socializing of residents. The social function of parks is supported by their proximity to houses. There are car-free play streets overseen by surrounding houses to provide both safety and security for children playing. Vauban is connected by light rail and bus, as well as by walking trails and cycling paths, to the rest of Freiburg and its main train station [48].

#### Greater Vancouver, Canada

Greater Vancouver provides an example of measures to achieve characteristic 2 of a healthy city: “An ecosystem which is stable now and sustainable in the long term”. Metro Vancouver is an inter-municipal arrangement with a focus on urbanization management, mass transit, waste management, and protection of natural resources and habitats [55]. It adopted the Livable Region Strategic Plan in 1996. The Plan had a principal component of the Green Zone which designated areas with great ecological value for protection from urbanization. The aim was to prevent the negative consequences associated with urban sprawl, both in terms of ecological impact and social issues. It sought to reduce pressures to convert green areas to urban uses and enable an efficient public transit system [56]. The Plan has helped to accommodate an additional 1,000,000 people in the metropolitan area over the past 30 years without compromising productive farmland, habitat, and important green space [50].

The joint work of cities in Greater Vancouver to reduce car transportation over long distances and air pollution and protect water basins and regional parks from urban expansion had multiple health benefits [57]. These health benefits were not only long-term. According to interviews with medical experts, Shore [58] suggested that a reduction in air pollution contributed to saving the lives of those who contracted coronavirus in the region during its pandemic. This is because lower levels of nitrogen dioxide and delicate particulate matter in the air helped their respiratory system to recover [58].

#### Singapore (City-State)

The city-state of Singapore applies housing and school policies to achieve characteristic 3 of a healthy city: “A strong, mutually-supportive and non-exploitative community” [72]. Between 1961 and 2013, the public housing authority constructed over one million high-rise housing units, which provided accommodation for approximately 90% of the population consisting of citizens and permanent residents. Additionally, more than 85% of households within these residences were homeowners [73]. Singapore commits to universal housing provision for all citizens. The owner of a public housing flat can sell the flat at market value, realizing nontaxable capital gains toward payment for a higher quality, larger flat. This effective provision of public housing has allowed the government to intervene in other aspects of social life by tying respective social policies to housing policy. These social interventions aim to nurture individuals who possess empathy, participate responsibly, and exhibit sensibility in a society that is diverse in terms of ethnicity, culture, and religion [74]. To support this objective, a social mix in public housing estates is planned, particularly among the three major ethnic groups, namely Chinese, Malays, and Indians. The first-come-first-served housing allocation rule acts to randomly distribute the three major races [61]. The intention is to ensure an ethnic mix in every housing block [72]. However, this does not prevent minority groups from applying for housing in areas in which they were traditionally concentrated [61]. Also, community gardens in public residential estates bring residents of different ethnic backgrounds together for shared management and utilization of these fruitful gardens. There is also green space between housing blocks fitted with furniture and equipment for different ages [63].

#### Seattle, WA, USA

Seattle provides an example of measures to achieve characteristic 4 of a healthy city: “A high degree of public participation and control over the decisions affecting one’s life, health and well-being”. In the 1990s, the City of Seattle adopted an initiative to take public engagement to its highest form, citizen control [84]. The background to this initiative of Seattle was, however, oppositional. The story commenced with the enactment of Washington State’s Growth Management Act in 1990. In response, Seattle developed a revised Comprehensive Plan to conform to this act. However, the introduction of this new plan was met with resistance from local neighborhood groups within Seattle. Consequently, as an attempt to mitigate these concerns and foster community involvement, Seattle implemented a neighborhood planning program in 1994 [85].

In this program, 38 neighborhoods have developed individual plans to align with the Comprehensive Plan while catering to the specific requirements of each community. This approach was specifically designed to be driven by the community, ensuring active participation from both residents and City staff. Moreover, it aimed to promote a collaborative environment where citizens and City officials learn together and develop harmonious ways of improving living conditions [86].

By 2006, Seattle’s neighborhood plans had been integrated into the city’s planning system for 8 years. The implementation progress of these plans was significant, with 80% of the recommended actions being completed and a funding amount exceeding $700 million successfully acquired. The involvement of key departments and developers in consulting the neighborhood plans before making decisions demonstrated their recognition and respect for this grassroots-level initiative. Furthermore, resident groups relied on these plans as a reference when embarking on new projects within their neighborhoods. It is important to note that developing and executing these neighborhood plans required collaborative efforts from approximately 30,000 individuals including residents, city officials, local business representatives, developers, and politicians. Much of what the neighborhood plans imagined can be seen today. These include new clinics, redesigned streets and safer sidewalks, libraries, schools, community centers, parkland, public art, revitalized historic business areas, and new cultural centers [80].

#### New York City, USA

New York is an example of a city adopting some measures to achieve characteristic 5 of a healthy city: “The meeting of basic needs (food, water, shelter, income, safety, work) for all the city’s people”. New York is a wealthy city, but homelessness, drug addiction, poverty, and ill health have been notable among particular social groups. In response to these issues, the City launched a program of “public housing that worked” [98]. The program housed over 400,000 tenants in large multi-family apartment complexes. These apartments apply management methods of middle- and upper-class buildings for poor and working-class populations to ensure that these apartments remain decent and do not become tenements [98]. However, there were people who, because of their mental health issues, addiction, and other lifestyle problems, did not qualify for this public housing. The only available shelter for these people could have been collective, temporary, and supervised accommodations. However, New York’s *Pathways to Housing* program provided immediate access to independent scatter-site apartments for individuals with psychiatric disabilities and substance addiction [99]. As a condition for receiving housing, clients are not obligated to engage in substance abuse or psychiatric treatment. Following the placement of housing, an assertive community treatment team provides ongoing assistance with substance abuse treatment, psychiatric and general medical care, and vocational services [91].

New York has taken steps to help people in low-income neighborhoods meet their need for fresh food. According to research conducted for the Mayor’s Food Policy Task Force, it was found that several low-income neighborhoods in the City have limited access to fresh food stores. This lack of affordable and fresh food options in these areas has been associated with elevated rates of diet-related diseases such as heart disease, diabetes, and obesity. To address this issue, Food Retail Expansion to Support Health Zones (FRESH) was established. They provide incentives for grocery stores in low-income neighborhoods to supply fresh products to support the nutrition of residents [88]. For example, the FRESH program gives property owners the right to construct larger buildings with reduced parking requirements if they include a FRESH supermarket [100].

#### London, UK

London provides an example of measures to achieve characteristic 6 of a healthy city: “Access to a wide variety of experiences and resources with the possibility of multiple contacts, interaction and communication”. Most neighborhoods provide walking access to shops, workplaces, community spaces, and parks. However, there are steps to ensure that wider experiences are also rich and accessible for the citizens. One example is seen in London’s Green Belt. New woodlands are set for creation in the Green Belt to ensure that it provides a contrasting natural experience. Public transport to the Green Belt is set to improve to ensure that the Green Belt better serves social and mental health purposes [112]. Access to the Green Belt is easy with the Oyster card. It is a single payment method for all modes of public transport in London and certain areas around it. Public transport is seamlessly connected between its modes (e.g., bus, underground, train) and with footpaths, bike share docking stations, and car/taxi pick-up and set-down points [113].

London’s measures to support the mobility of people with special needs are noteworthy. Before the 2012 Games, the Mayor pledged to make London more accessible for both disabled visitors and Londoners. A whole range of measures were put in place to achieve this, including measures to enable step-free journeys for wheelchair users [114]. Older citizens are another group that receives mobility support. They are offered free access to a relatively extensive public transport network through a Freedom Pass. Aside from enhancing their accessibility to health-related goods and services, this pass also contributes to the mental well-being of older citizens. It enables them to regularly travel by public transport which gives them opportunities for multiple social interactions on the public transport journey to overcome chronic loneliness and maintain their mental health [169].

#### Nantes, France

Nantes is known for its measures to achieve characteristic 7 of a healthy city: “A diverse, vital and innovative city economy”. The City of Nantes and its integrated metropolitan area have been leading the continuous growth of a multi-sector, localized, and knowledge-based economic cluster [121]. Cities of the metropolitan area work together in their association, *Nantes Métropole*, to infrastructure their joint economic cluster and attract more complementary activities [122]. The City of Nantes is a hub of banking, sales and retailing, health sector, and technology start-ups. Most of these activities and the lives of their employees are based in the city center [123]. Other cities in the metropolitan area have complementary manufacturing and agriculture sectors. This metropolitan economic cluster brings multiple benefits to the health of citizens beyond providing employment and income.

For example, the Nantes-based collective *Makers For Life* has been working alongside academic partners and hospitals, the plastic sector, numerous companies (e.g., Clever Cloud, Oxygen Ouest, Le Palace), local authorities, and others to create MakAir. It is an artificial ventilator made for hospitalized patients with coronavirus [124]. In the same context of cooperation with various local partners, Nantes-based laboratory *Xenothera* introduced a new coronavirus disease treatment designed to stop patients from becoming seriously ill [125]. Disruptions in the global supply of food products during the coronavirus pandemic were less impactful in the Nantes metropolitan area. This was because of the agriculture sector in the metropolitan area which also acted as a solidarity sector in this period. For example, with the help of some volunteer farmers, an initiative called *Nourishing Landscapes* transformed 50 green municipal sites in the City of Nantes into fruitful vegetable gardens. This was intended to feed the economically vulnerable during the pandemic [162].

#### Exeter, UK

Exeter provides an example of measures to achieve characteristic 8 of a healthy city: “Encouragement of connectedness with the past, with the cultural and biological heritage and with other groups and individuals”. The urban regeneration of Exeter’s Historic City Centre used heritage in producing everyday social life. It opened fresh street views of the historic Cathedral and created everyday contact settings for the medieval Almshouses and the City Wall [135]. The remnants of the Roman/medieval City Wall became part of a new pedestrian route and shopping street connecting public spaces, such as the plaza and the pocket park. This everyday heritage route is called the City Wall Trail. It connects residents with tourists in multiple ways, for example, in the pocket park that has space for lunch and relaxing between apartment houses and the Wall [132].

The heritage entrenched in Exeter’s urban layout is not only a prominent tourist attraction but also part of an everyday commute that provides spontaneous contact for many residents. It stimulates conversations between people of different ages, roots, and groups about the Wall, Cathedral, etc., as they travel to work, school, or shops. This everyday heritage design helps improve place identity and social bonds that are important for mental health and for the availability of mutual support during times of crisis [135].

#### Copenhagen, Denmark

**Fig. 5:**
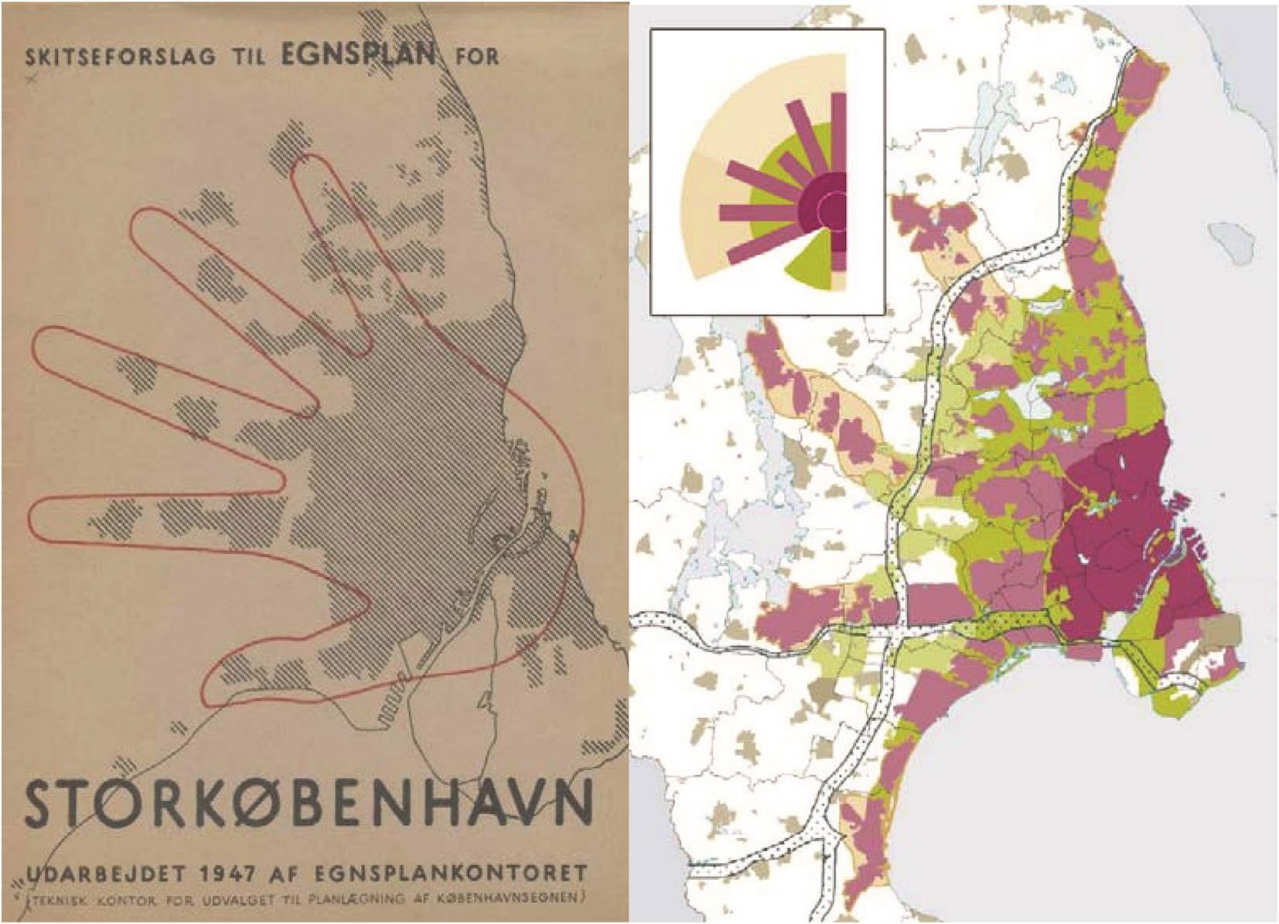
Copenhagen Five-Finger Plan 1947 version (left) and 2007 version (right) [146]

Copenhagen is known for its measures to achieve characteristic 9 of a healthy city: “A city form that is compatible with and enhances the above parameters and behaviours”. The Copenhagen metropolitan area has avoided both urban sprawl and overly dense urbanization that lowers the quality of life. This was made possible with the design of a modular urban development in a so-called “Five-Finger Plan.” The Plan was drafted in 1947 for the greater Copenhagen area with support from local governments of the area. The Five-Finger Plan still acts as a basis for the integration of local development plans in the area (Fig. 5) [144]. The Five-Finger Plan proposed to carry out urban expansion along five commuter rail lines radiating from the dense urban texture of central Copenhagen (palm) to the surroundings. Most public buildings and high-density residential zones are concentrated around rail stations, while residential neighborhoods exhibit a moderate level of density [146]. The areas between the five corridors (fingers) serve as green wedges. In these areas, the construction of neighborhoods and shopping malls is prohibited [146]. This is because these green areas are designated for agriculture and healthy pleasure of outdoor activities in proximity to the residential neighborhoods [147]. However, fingers are modular and consist of sub-modules or joints. This means that fingers can be extended by new neighborhoods or shortened, and new fingers can be added [144].

#### Washington, DC, USA

Washington, DC applies a range of measures to achieve characteristic 10 of a healthy city: “An optimum level of appropriate public health and sick care services accessible to all”. Some of these measures might be like those applied in many other cities. One example is “Shortage Designation” to prioritize the allocation of healthcare resources. A Shortage Designation can refer to a shortage of healthcare providers available to the general population in an area. Or it can refer to a shortage of healthcare providers available to a specific population that faces economic, cultural, or linguistic barriers to receiving healthcare in an area. The designation is evaluated and updated every 3 to 5 years through the meticulous gathering of detailed information regarding healthcare practitioners practicing in Washington, DC. [161].

**Fig. 6:**
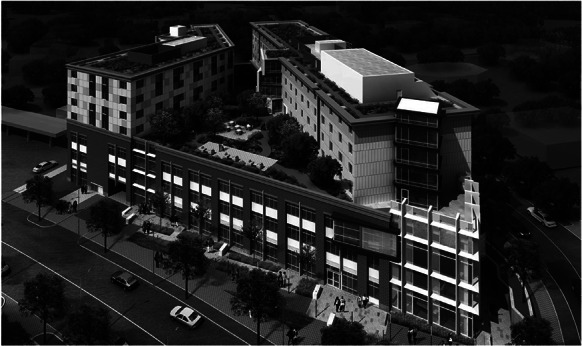
The Conway Center in Washington, DC [8]

Some other measures to improve health service for underserved communities are more specific in Washington, DC. As an illustration, the Conway Center (Fig. 6) serves as a prime example of how multiple aspects that influence health can be effectively addressed within one building complex. Located in the historically neglected Northeast Washington, DC area, this facility integrates various measures to promote health and provide healthcare. This includes the provision of a community clinic catering to approximately 15,000 low-income patients annually, alongside over 200 apartments for low-income families or individuals transitioning from homelessness into permanent supportive housing. Moreover, the Conway Center provides amenities such as a green roof with communal spaces and playgrounds for children. Additionally, it has a job training center and provides office and retail space. Furthermore, its proximity to a subway station just across the street enhances accessibility for residents by offering transportation options that connect them to employment opportunities and essential resources located throughout the city [8].

### Healthy Sectors in a Healthy City

Healthy city measures and their examples indicate that a healthy city is a system of healthy sectors. This subsection synthesizes the study results in terms of healthy directions for nine sectors in a healthy city. It could guide the strategies of these sectors in integration with urban health promotion.

#### Transportation

Healthy transportation involves the integrated promotion of physical activity, environmental protection, ease, safety, and equity in access to a wide variety of resources and contacts in and around cities.

#### Housing

Healthy housing involves affordable shelter that also supports other needs (such as the need for work, belonging, contact with nature, and healthcare services) with minimum environmental impact. It is adaptable to the changing needs of users to provide lifelong satisfaction.

#### Schools

Healthy schools seek to keep classmates from the whole of the community together from the first grade through high school. They connect students with their environmental and cultural roots and promote participation and healthy lifestyles in the context and delivery of education.

#### City Planning

Healthy city planning mediates between different stakeholders and sectors to stimulate urban development that supports the multiple determinants of health. It promotes a dense urban form that is scalable, and livable and affordable for citizens. Healthy city planning involves an integrated focus on neighborhoods, the city center, and the region to support health determinants from shelter to food, income, and contact with others and nature, but it ensures that different areas in and around the city maintain their particular character and specific relationship with the environment.

#### Local Government

A healthy local government has freedom from central interference but the ability to provide effective services in consultation with citizens. Its citizen consultations improve health in multiple ways, for instance, by giving information, voice, and influence to citizens.

#### Environmental Management

Healthy environmental management involves a systematic mechanism of impact assessment. It considers the impacts of developments in direct terms and by cumulative disruptions to environmental and social systems. It applies the impact assessment of policies in impact assessment of projects and includes 'no project option' in impact assessment.

#### Retail

Healthy retail should support meeting basic needs for goods, income and work. It should enable street vending where contributing to meeting these needs. Healthy retail should support cheap eating places, farmers markets, fresh food stores, and small service establishments. 

#### Heritage

A healthy city reflects the character of heritage in development, integrates heritage protection with economic health, and connects heritage with the everyday life of citizens.

#### Healthcare

Healthcare in a healthy city is community-sensitive and *actually* accessible. Communities participate in setting the objectives and design of healthcare services. Neighborhood clinics provide services tailored to the health map of local communities and their cultural and economic conditions. Policies about the number and locations of public healthcare facilities seek to equalize the realized access of citizens to healthcare.

### Gaps in the Literature

This study found that the literature about measures to achieve the characteristics of a healthy city is overall rich. However, this is less applicable to characteristic 5 (i.e., the meeting of basic needs for all the city’s people) and characteristic 10 (i.e., public health and sick care services accessible to all) of a healthy city. Particularly, informal and intersectoral measures that are critical to achieving these characteristics need more research. For example, we could not find cases in the literature where action is taken as part of a healthy city program to enable street vending. Likewise, there is limited evidence about intersectoral measures to integrate healthcare services with houses and communities.

## Conclusions

This scoping review aimed to answer the following question: what systematic measures are needed to achieve a healthy city? A set of measures were extracted from the literature and presented in 29 themes for application by diverse professional actors shaping our cities as a whole. A synthesis of the findings identified healthy directions for nine sectors in a healthy city. 

This scoping review applied a robust methodology and covered a vast comparative literature from different fields and contexts. However, discretion in applying the measures identified in this study is advised because different contexts might require different healthy city approaches. Meanwhile, the sectoral health directions synthesized in the study are more generalizable. Another consideration in applying the findings of this study is that, as a scoping review, this study did not perform a formal assessment of the methodological quality of the included literature records. This is a general limitation of scoping reviews. However, most included records were peer-reviewed studies, and the charted measures were contents reflected in more than one record. Another limitation of this study is subjectivity, which is inherent to such qualitative research. However, we took steps to acknowledge and minimize subjectivity in the protocol and conduct of this systematic scoping review as well as its inductive content analysis. These steps, that were mentioned in the Section “Research Method” of the paper (e.g., testing the sample quality and refining the unrepresentative sample), helped avoid systematic and significant bias in the study. Meanwhile, there is always a possibility of missing some important studies in scoping reviews of enormous literature like that of healthy cities.
